# Quality Classification of Injection-Molded Components by Using Quality Indices, Grading, and Machine Learning

**DOI:** 10.3390/polym13030353

**Published:** 2021-01-22

**Authors:** Kun-Cheng Ke, Ming-Shyan Huang

**Affiliations:** Department of Mechatronics Engineering, National Kaohsiung University of Science and Technology, Kaohsiung City 824, Taiwan; kcke@nkust.edu.tw

**Keywords:** injection molding, cavity pressure curve, machine learning, multilayer perceptron neural network, quality indices, quality control

## Abstract

Conventional methods for assessing the quality of components mass produced using injection molding are expensive and time-consuming or involve imprecise statistical process control parameters. A suitable alternative would be to employ machine learning to classify the quality of parts by using quality indices and quality grading. In this study, we used a multilayer perceptron (MLP) neural network along with a few quality indices to accurately predict the quality of “qualified” and “unqualified” geometric shapes of a finished product. These quality indices, which exhibited a strong correlation with part quality, were extracted from pressure curves and input into the MLP model for learning and prediction. By filtering outliers from the input data and converting the measured quality into quality grades used as output data, we increased the prediction accuracy of the MLP model and classified the quality of finished parts into various quality levels. The MLP model may misjudge datapoints in the “to-be-confirmed” area, which is located between the “qualified” and “unqualified” areas. We classified the “to-be-confirmed” area, and only the quality of products in this area were evaluated further, which reduced the cost of quality control considerably. An integrated circuit tray was manufactured to experimentally demonstrate the feasibility of the proposed method.

## 1. Introduction

Injection molding has been extensively used for the mass production of components in various industries, including the automobile, construction, electronics, household appliance, medical equipment, and optics industries. The injection molding process must be rapid and reliable in the production of high-quality parts. However, several factors affect the quality of injection-molded parts, such as changes in raw materials, plasticizing quality, process parameter settings, and machine performance [1,2]. Currently, even the use of high-precision injection molding machines with sophisticated control algorithms cannot guarantee the production of parts with zero defects. The available machine process data cannot capture changes in the properties of the applied raw material, which can affect the quality of the components. Therefore, algorithms for predicting changes in materials cannot meet the required accuracy levels. The primary reason for this is that the flow behavior of polymer melts in a mold cavity is not adequately understood. Because of advancements in sensing technology, pressure and temperature sensors are available for detecting changes in the properties of polymer melts in molds and for monitoring and controlling the injection molding process [3,4,5,6,7,8]. Although the cost of installing cavity sensors in production systems is higher than the cost of using machine profiles, the use of cavity profiles helps to reduce prediction errors [9,10]. A high correlation exists between part quality and features extracted from cavity pressure curves [11,12]. In addition, quality indices can help to rapidly determine the quality of parts without the need for any measuring equipment, a process that is feasible for quality control in actual injection molding operations. Although injection-molded parts are not without defects, their quality has improved considerably because of the aforementioned advancements.

Artificial neural networks (ANNs) that can fit input and output data that exhibit a highly nonlinear relationship have potential applications for automatic quality prediction and process parameter optimization in injection molding. Several studies have been conducted on the use of ANNs in injection molding. For example, Zhou et al. [12] used an autoencoder model to automatically extract cavity pressure characteristics from a cavity pressure curve; thus, they successfully interpreted the physical relationship between cavity pressure and part quality. Oliaei et al. [13] used Taguchi orthogonal arrays to design experiments to change the mechanical setting, such as the mold temperature, holding time, holding pressure, and barrel temperature; they applied an ANN to predict the shrinkage and warpage of the injection-molded parts. In their study, to obtain a model that satisifies high training accuracy, a long training time is required. Ozcelik and Erzurumlu [14] used analysis of variance, an ANN, and a genetic algorithm to eliminate warpage in plastic injection molding, which took about 3 h in model training. Reddy et al. [15] used commercial simulation software to analyze shrinkage and warpage values and then employed a multilayer perceptron (MLP) backpropagation algorithm to predict the shrinkage and warpage values, percentage deviation, and average percentage deviation. Yin et al. [16] proposed a method for predicting the warpage of injection-molded parts and optimizing them using commercial finite element simulation software, an orthogonal experiment, and a backpropagation neural network, which required 30 min of training on the workstation. Bensingh et al. [17] used a backpropagation ANN with particle swarm optimization to generate optimal process parameters for manufacturing an aspheric lens. Guo et al. [18] used input and output data obtained from finite element simulations to train various neural networks to function as warpage prediction models; they concluded that a hybrid particle swarm optimization–backpropagation algorithm is the ideal model for predicting the warpage of microcellular foaming material. A feedforward MLP neural network model has been proven to be suitable for the regression and classification of data in applications such as quality inspection and has been used in many studies [9,19]. The main advantage of the MLP model is to solve complex nonlinear problems, such as classification; however, the training process is time-consuming. For model training, how to use appropriate input is not only related to the length of training time, but also to the accuracy of training. In particular, most previous studies have focused on using process parameters as input values for model learning, which may not be appropriate. Instead of using process parameter settings as input values, quality indices that are highly related to part quality can provide sufficient information for model learning, which has attracted attention recently [9,10,12].

The present study aimed to apply an MLP model and quality indices extracted from pressure curves to develop a model for automatically and rapidly determining the quality of injection-molded parts. By adopting highly relevant quality indices, filtering erroneous outliers from the data, and converting measured qualities into grades, we reduced the size of training data; thus, the model training process could be expedited, and high prediction accuracy could ultimately be achieved. In addition, the MLP model provides inspection results that are “confirmed” and need “to-be-confirmed”; operators must pay attention to the inspection results that need to be confirmed in order to ensure efficient quality control.

## 2. Methodology

### 2.1. Quality Indices

To predict the quality of a part with respect to changes in molding conditions, we evaluated various quality indices. Quality indices that are highly correlated with part quality are used as input parameters for model training in order to increase prediction accuracy. By referring to the pressure information, the following quality indices were selected [9].

First-stage holding pressure index $\left(Ph_{index}\right)$: This represents the average holding pressure in the first stage. The holding process, which is also called postfilling, involves compensating for the cavity gap caused by polymer melt shrinkage. Notably, holding pressure is critical to the geometric quality of molded parts.

Peak pressure index ($Pp_{index}$): This represents the maximum pressure during filling and compression. In injection molding, pressure is applied to drive the polymer melt into the cavity. The maximum pressure affects the amount of polymer melt entering the cavity; thus, the maximum pressure determines the geometric quality of the injection-molded part.

Residual pressure drop index ($Pr_{index}$): This represents the average residual pressure drop in the cavity during cooling. The average residual pressure drop is related to the residual stress in the processed polymer. A high average residual pressure may cause geometric warping, and a low average residual pressure may engender an undersized molded part.

Pressure integral index ($PI_{index}$): This represents the integral of the pressure curve with time over a molding cycle (i.e., from filling to compression, holding, and, finally, cooling). This index is related to the total pressure characteristics of the polymer melt during the injection molding process. The variation in $PI_{index}$ may reflect the change in part quality, particularly the change in part weight [9].

These four indices have different ranges of values and are normalized between 0 and 1. Using these indices as inputs for model training could engender rapid convergence and high accuracy.

### 2.2. MLP Models

An MLP model, a supervised ANN learning model typically comprising an input layer, hidden layers and an output layer, was used in this study [9]. Equations (1) and (2) present the expressions of the sigmoid function used in the hidden layers, and Equation (3) presents the expression for the softmax function used in the output layer. In binary classification, the sigmoid function (also called a logical function) maps the summary of the input function to the interval (0, 1). The softmax function (also called the soft-argmax or normalized exponential function) normalizes an input vector of $N_{L}$ real numbers to a probability distribution that comprises $N_{L}$ probabilities proportional to the exponentials of the input numbers. The softmax function normalizes components to the interval (0, 1), with the total value being 1. Large input components correspond to large probabilities. Thus, the softmax function maps the nonnormalized output of a network to a probability distribution over the predicted output classes.

(1) $$\varphi_{1}\left(o_{i}\right)=\frac{1}{1+e^{-o_{i}}}$$

(2) $$\varphi_{2}\left(o_{i}\right)=\frac{e^{o_{i}}}{\sum_{j=1}^{N_{L}}e^{o_{j}}}$$

(3) $$\sum_{i}\varphi_{2}\left(o_{i}\right)=1$$

We used the accuracy function Ai given in Equation (4) to evaluate the convergence of model training. By comparing the predicted value with the actual output value, the accuracy of the training can be evaluated. For example, if the predicted value is consistent with the actual value, the weighting value is recorded and training is continued for the next dataset; otherwise, the weighting value is adjusted. The terms $N_{mis,i}$, $N_{total}$, and $N_{train}$ in Equation (4) represent the number of misclassified datapoints at the *i_th_* training iteration, total number of datapoints, and total number of training iterations, respectively. The accuracy function converges as the number of training iterations increases. By setting the stopping criteria, the quality of model training can be obtained.

(4) $$A_{i}\;=\left(1-\frac{N_{mis,\;i}}{N_{total}}\right)\times 100\%$$

The number of hidden layers and neurons will affect the convergence of model training. An MLP model with a large number of neurons and layers requires a large number of calculations on the weighted value, which may cause a difference between the predicted value and the actual value. In contrast, MLP models with few neurons or layers may not be able to establish a good connection between the input layer and the output layer; therefore, the values predicted by these models may be inaccurate. In addition, erroneous data from experiments may cause incorrect results in model training. Therefore, outliers filtering must be performed before model training to ensure the prediction accuracy of the trained model.

### 2.3. Outlier Filtering

We used standard scores (also called z-scores, *z*) to identify outliers. The z-score, as shown in Equation (5), is dimensionless; *x*, *μ*, and *σ* represent the original score, average score, and standard deviation from the experiment, respectively.

(5) $$z=\left|\frac{x-\mu }{\sigma }\right|$$

**Figure 1:**
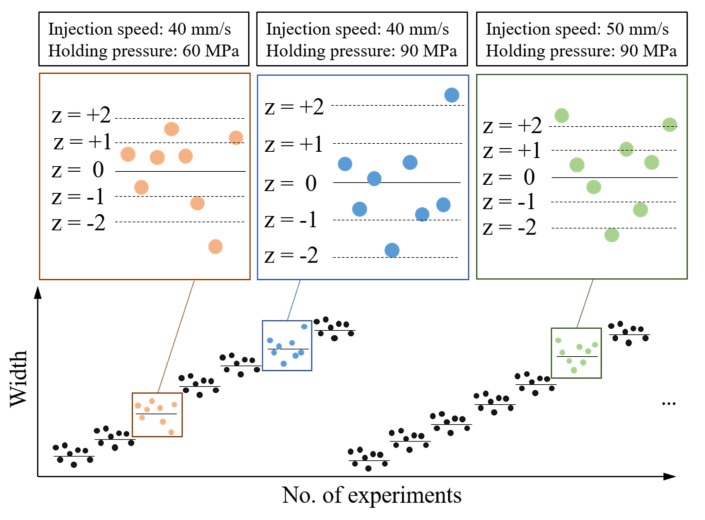
Example of z-score outlier filtering.

The standard score represents the deviation between the original score and the average value of the population and is calculated in terms of standard deviation. The standard score denotes the number of standard deviations from the point of interest to the average. Figure 1 illustrates the process of filtering outliers from each experimental dataset for the same process parameter settings. Consider, for example, an injection speed of 40 mm/s and holding pressure of 60 MPa; if the z-score is set to 2, then datapoints outside the z-score range [−2,2] can be regarded as outliers. The lower the z-score is, the higher the number of outliers. However, excessive filtering of datapoints may result in a loss of important information, causing a relatively low MLP training performance. Therefore, we used the z-score as a boundary to eliminate outliers, retaining informative data to achieve stable model training.

### 2.4. Quality Classification

To classify the quality of injection-molded parts, we converted the measured qualities (such as geometrical width) into multiple grades. As indicated in Equation (6), we aggregated the data into *N_g_* grades evenly spaced between the minimum and maximum values in *W*. Δ*W* represents the bandwidth of each grade. Therefore, the grade location *X_u_* of *W_j_* is for the output data from MLP model training. In this study, we evaluated the influence of grade numbers (such as 5, 10, 20, and 50) on training accuracy and width prediction accuracy. For example, an increase in the grade number means that the bandwidth of each width grade will be decreased, resulting in poorer training accuracy in the MLP model; a decrease in the grade number will increase the bandwidth of each width grade, leading to a decrease in the resolution of width prediction. Therefore, it is best to optimize the choice of grade number.

(6) $$N_{g}=\frac{\underset{j}{\max}W_{j}-\underset{j}{\min}W_{j}}{\Delta W}$$

*N_g_* determines the prediction error of the MLP model; specifically, the maximum error in the prediction of the quality of injection-molded parts is Δ*W*. In theory, a higher *N_g_* value implies a lower prediction error. However, this value is related to the actual measurement accuracy achieved by the device. In addition, a lower *N_g_* value may lead to poor training accuracy for the MLP model. In this study, we evaluated various Δ*W* values to obtain the optimal value for predicting the geometric quality of injection-molded parts.

## 3. Experimental

**Figure 2:**
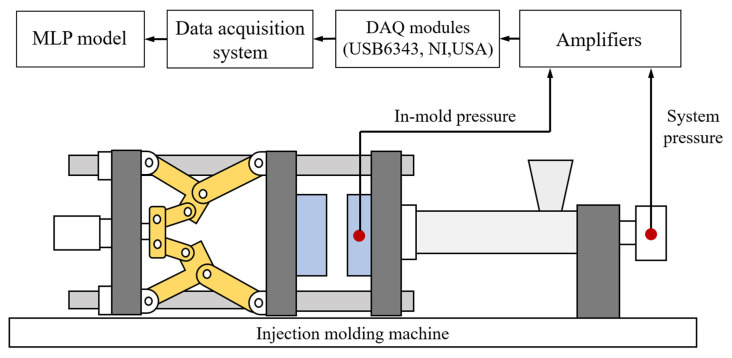
Schematic of the quality inspection system.

Figure 2 presents a designed MLP-based quality inspection system comprising an injection molding machine, an injection mold for manufacturing integrated circuit (IC) trays, a data acquisition module, two types of pressure sensors, and a computer for MLP modeling. Each component is detailed in the following sections.

### 3.1. Injection Machine, Material, Mold, and Sensor

**Figure 3:**
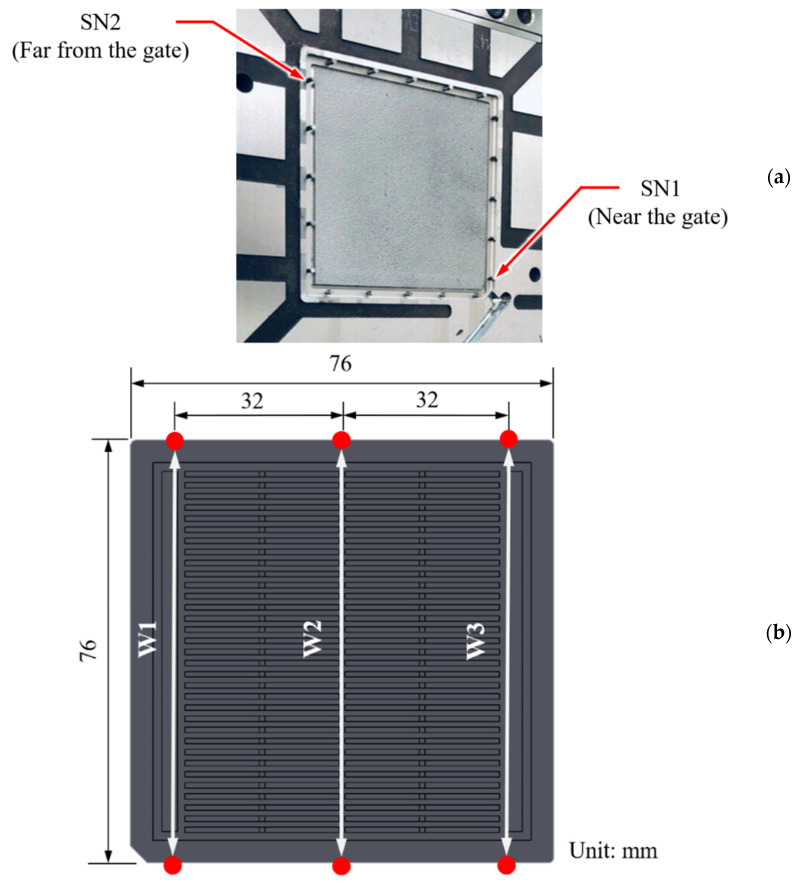
Positions of (**a**) pressure sensors and (**b**) part widths measured by coordinate measuring machine.

We manufactured IC trays by using an all-electric-driven injection molding machine with a clamping force of 100 t (CT-100, Fu-Chun Shin Corporation, Tainan, Taiwan). The polymer used was an acrylonitrile–butadiene–styrene copolymer (PA-756 Chi-Mei Corporation, Tainan, Taiwan). The ratio of the flow distance to the average tray thickness was 124. The length, width, and thickness of the IC tray were 76, 76, and 4.4 mm, respectively. To investigate the overall flow state of the melt and the quality indices of melt, we installed two types of pressure sensor in the mold—one near the gate (SN1) and one far from the gate (SN2). Figure 3a shows the location of each sensor used in this study. Assuming that the polymer melt will exhibit a laminar flow and because the ratio of the flow distance to the average thickness is high (124); accordingly, the pressure change along the thickness direction is ignored. To sense cavity pressure signals, pressure sensors (SSB01KN08X06H and SSB01KN08X08H, Futaba Corporation, Mobara, Japan) were mounted on the back of the ejectors and the pressure signals are extracted by the DAQ module (USB6343, National Instrument, Austin, USA). The sampling frequency and recording time of each shot are 1000 Hz and 35 s, respectively. That is, the datapoints of each in-mold pressure curve and system pressure curve are 70,000 and 35,000, respectively (i.e., the number of data points at each shot is 105,000). Figure 3b shows the geometry of the IC tray manufactured in this study. The W1, W2, and W3 of the manufactured part are considered as the key qualities and were measured using a precise coordinate measuring machine (CRYSTA-Apex S700, Mitutoyo Corporation, Kawasaki, Japan).

We executed a two-factor full-factorial experiment. Table 1 lists the process parameters for the injection molding of the IC tray. The injection speed ranged from 40 to 120 mm/s, and the first-stage holding pressure ranged from 40 to 100 MPa. This study aims at a wide range of process parameter adjustments, explores the correlation and outlier between quality indicators and quality (width), and then builds an MLP neural network model to discuss the feasibility of width prediction. The number of combinations of the various parameters was 63, and the number of samples for each combination was 6–8. After each shot, a system pressure curve and two cavity pressure curves (near-gate cavity pressure curve, SN1, and far-gate cavity pressure, SN2) were recorded.

**Figure 4:**
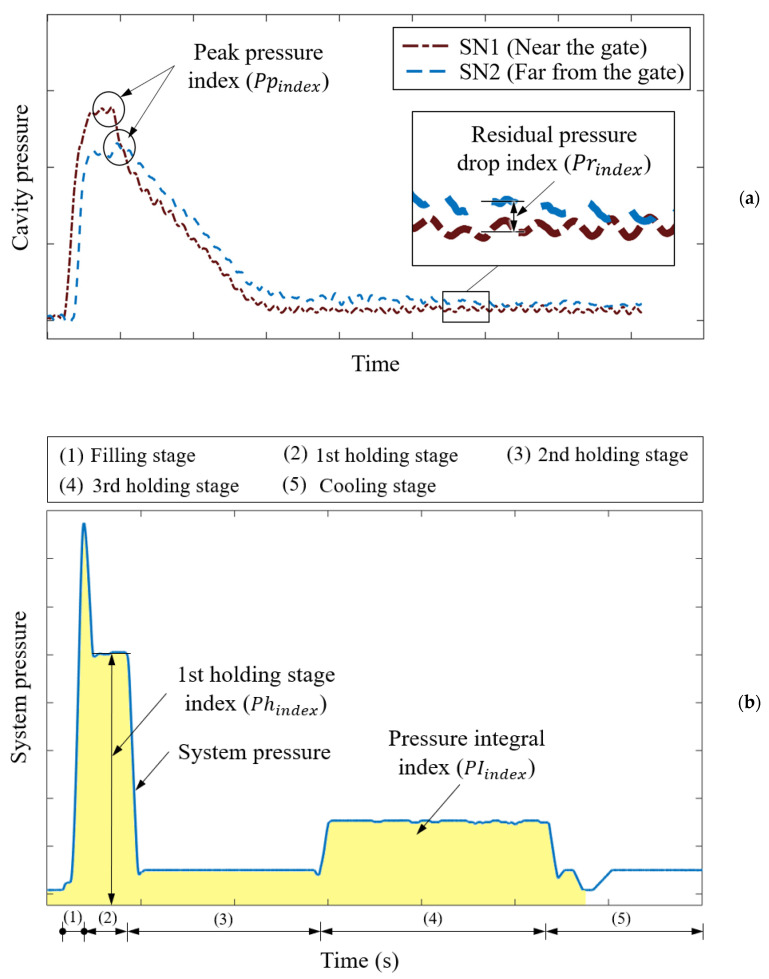
Quality indices: (**a**) peak pressure index ($Pp_{index}$) and residual pressure drop index ($Pr_{index}$ ); (**b**) pressure integral index ($PI_{index}$ ) and first-stage holding pressure index $\left(Ph_{index}\right)$.

The system pressure curve was used to obtain two quality indices: *Ph_index_* and *PI_index_*. SN1 was used to obtain *Pp_index_*, and SN2 was used to obtain *Pr_index_*. Figure 4a illustrates the physical meaning of $Pp_{index}$ and $Pr_{index}$ in the cavity pressure profiles obtained using the two sensors. $Pp_{index}$ represents the peak pressure in the filling and holding stages. This maximum pressure indicates the degree of compression on the polymer melt and determines the geometric quality of the injection-molded part. $Pr_{index}$ represents the residual pressure drop in the cooling stage. This pressure drop indicates local shrinkage. The system pressure (Figure 4b) provides the driving force for the polymer melt to overcome resistance during mold filling and compression; thus, the flow pressure of the polymer melt flowing through the pressure sensors varied according to the flow distance. Figure 4b shows the physical meaning of $Ph_{index}$ and $PI_{index}$. $Ph_{index}$ represents the main packing capacity in the holding stage, which is related to the molding weight and part geometry. $PI_{index}$ represents the momentum required for mold filling and compression; it indicates the quality of the injection-molded parts. These quality indices were the inputs for the MLP model for evaluating part quality; they were further evaluated using the Pearson correlation coefficient (PCC) [20].

As presented in Table 2, the PCCs for the four quality indices were high (i.e., >0.75). The total number of experiments conducted in this study was 504, with all four indices used in each subexperiment. The output data were the quality classification of three geometric widths (W1, W2, and W3) of the IC tray.

### 3.2. Outlier Filtering, and Quality Grading

Table 3 lists the results we obtained after filtering outliers from experimental datapoints by using various z-scores (1.5, 2, and 2.5); we observed no outliers when we applied a high z-score (e.g., 2.5). Conversely, a low z-score (e.g., 1.5) could exclude a considerable amount of information (the number of datapoints excluded as outliers was approximately 10% of the total number of datapoints). When we applied a z-score of 2, we observed 10–15 outliers for the three widths (i.e., the number of datapoints excluded as outliers was approximately 2% of the total number of datapoints). Specifically, as the standard score decreased, the number of outliers increased. However, excessive filtering of outliers may result in the loss of valuable data, which may affect MLP model training. Accordingly, the selection of the ideal z-score is an optimization problem that involves a trade-off between the filtering of incorrect data and model training accuracy. When the standard score was set to 2, the numbers of valid datapoints for W1, W2, and W3 were 494, 489, and 493, respectively. These datapoints were then divided into two groups, with 100 being used for model testing and the remaining being used for model training.

The numbers of grades for the three width qualities were set to 5, 10, 20, and 50. The number of grades typically determines the precision of the predicted width. Consider, for example, W1; when a z-score of 2 was used for outlier filtering, the maximum and minimum of experimental datapoints were 76.048 and 75.872 mm, respectively. The bandwidth of each grade was defined as the width range (i.e., the maximum width minus the minimum width) divided by the number of grades, which was 34.8 µm when the number of grades was 5. This value indicates the precision of the prediction model for the widths. Table 4 lists the bandwidths derived for the three widths. The highest precision was achieved when the number of grades was 50 (bandwidth of 3.5 µm). Because the width quality was classified into grades, the datapoints located between grades may lead to erroneous predictions owing to further MLP models, resulting in loss of quality control. Therefore, users can specify the ideal number of grades to strike a balance between MLP model precision and prediction accuracy. If only “qualified” and “unqualified” areas are considered in quality control, a user can define a “to-be-confirmed” area, which is located between the qualified and unqualified areas. Accordingly, only products in the to-be-confirmed area must be measured; this represents a low-cost method for ensuring high-quality parts.

### 3.3. MLP Model

**Figure 5:**
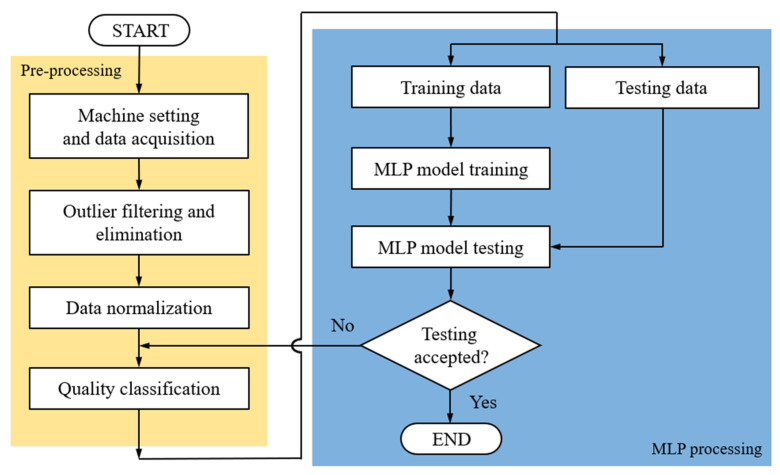
Flowchart of multilayer perceptron (MLP) modeling.

Figure 5 presents the flowchart of the operating procedures of the MLP modeling in the research. In the pre-processing stage, it contains four steps:

1. Machine setting and data acquisition: Use the two-factor experiment method to adjust machine settings, and perform data acquisition to capture different pressure curves for each shot. Then, convert the pressure curves into quality indices. The width values of the part will be measured by a high-precision coordinate measuring machine as the qualities used in the next step;
2. Outlier filtering and elimination: The database obtained through the experimental design method will be judged its abnormal state by standard scores, which are set to 1.5, 2.0 and 2.5, respectively. Once the dataset obtained from the same machine setting is higher than this setting, it is defined as an outlier and filtered out;
3. Data normalization: In order to reduce the influence of different data dimensions on convergence, this experiment normalizes the quality indices to an interval (0, 1);
4. Quality classification: The width values are converted to various grades (5, 10, 20, and 50 in this study).

- The MLP processing stage includes:

5. Training and testing data: A specific parameter group is selected as the input data, and the normalized data are divided into two groups, with 100 datapoints being used for model testing and the remaining datapoints being used for model training;
6. MLP model training: The MLP model in this experiment is built using modules in Python. The MLP model contains an input layer of four nodes; two hidden layers (one with 100 neural nodes and the other with 75 neural nodes); an output layer of 5, 10, 20, and 50 nodes which corresponds to various grades, respectively. Table 5 presents the hyperparameter settings for the experimental MLP model. The internal parameters of an MLP model are called hyperparameters: they indicate the feature settings of the training model [21,22] and include the number of iterations (epoch), batch size, number of hidden layers, number of neurons per layer, and learning rate. The optimization processing method is controlled by hyperparameter learning rate, and the lowest loss time function (when the value of loss function is lower than 1) is obtained under the experimental control as the basis. The training and testing (inferencing) time are less than 10 min and 0.003 s, respectively;
7. MLP model testing: When the model testing result meets the user-defined criteria (validation loss value is less than 1), the grade number was used to confirm the category of the predicted width. Otherwise, this step will return to quality classification and redo.

## 4. Results and Discussion

### 4.1. Effect of z-Score and Number of Grades on Training and Prediction Accuracy of MLP Model

**Figure 6:**
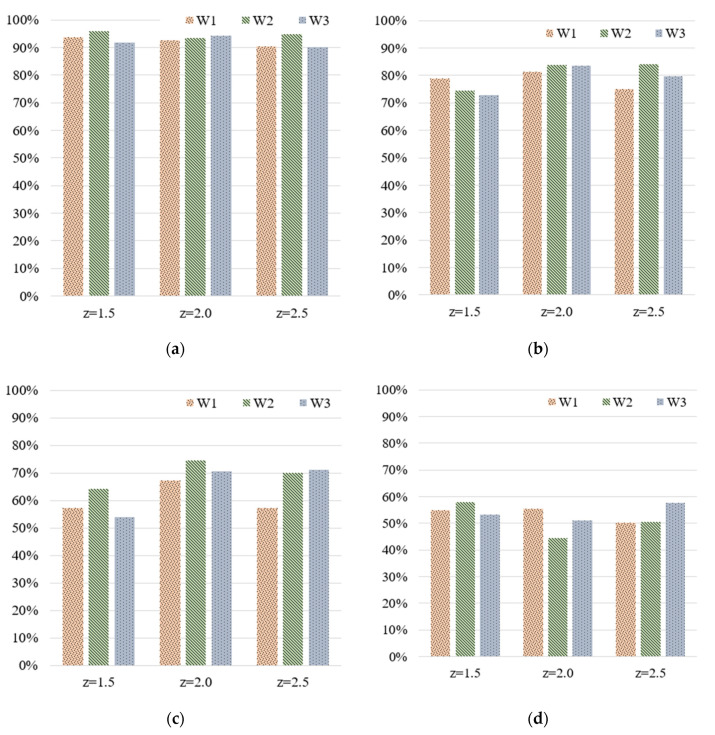
Training accuracy for the widths of the integrated circuit (IC) tray with respect to various z-scores and numbers of grades: (**a**) 5, (**b**) 10, (**c**) 20, and (**d**) 50 grades.

Figure 6 illustrates the model training accuracy levels for the three widths (W1, W2, and W3) at various z-scores. The deviation between the accuracy rates for the three widths was not high. Table 6 lists the deviations of the accuracy rates corresponding to the four z-scores; the average training accuracy rates corresponding to 5, 10, 20, and 50 grades were approximately 92–94%, 75–83%, 58–71%, and 50–55%, respectively. The average training accuracy rate for the various z-scores decreased as the number of grades increased. The training accuracy rate exceeded 90% only when the number of grades was 5. Therefore, the stipulated requirements could be met only when five grades were used. When the number of grades was low, the z-score did not have a considerable influence on the training accuracy. However, the use of a higher number of grades can provide more quality categories; therefore, the z-score was considered to affect the training accuracy. For example, when the number of grades was 10, the average training accuracy rates at z-scores of 2 and 2.5 reached 83% and 80%, respectively, which were higher than that (75%) obtained at a z-score of 1.5. Similarly, when the number of grades was 20, the average training accuracy rate at a z-score of 2 reached 71%, which was higher than those obtained with the other numbers of grades (58% and 66% for z = 1.5 and 2.5, respectively). Quality classification could not be performed when the number of grades was 50 owing to low training accuracy. Therefore, appropriate standard scoring could help the MLP model effectively filter outliers and obtain favorable quality predictions. However, excessive filtering may cause the deletion of normal data and loss of valuable information.

**Figure 7:**
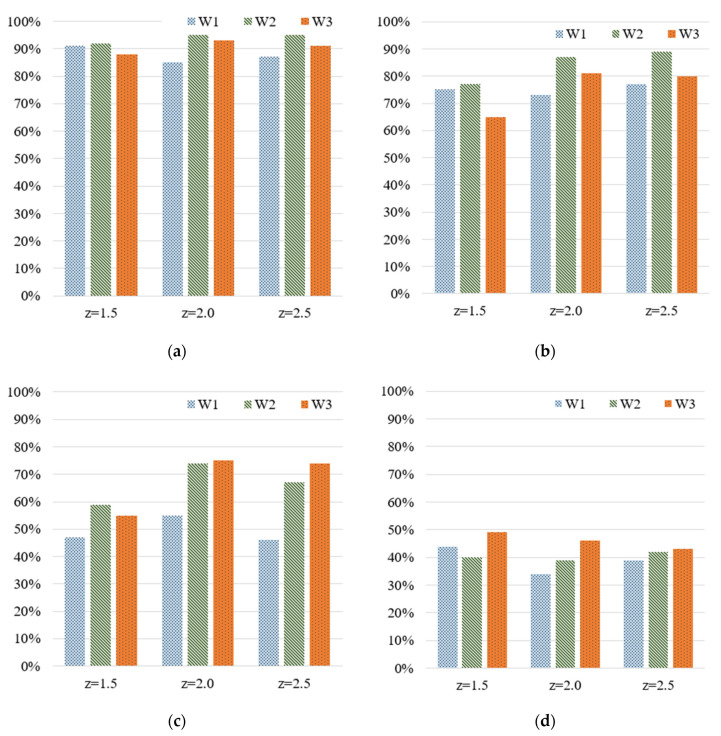
Testing accuracy for the widths of the IC tray with respect to various z-scores and numbers of grades: (**a**) 5, (**b**) 10, (**c**) 20, and (**d**) 50 grades.

Figure 7 shows the testing accuracy rates for the widths at various z-scores and numbers of grades. Table 7 lists the deviations between the accuracy rates corresponding to the four z-scores; the average accuracy rates observed when the numbers of grades were 5, 10, 20, and 50 were approximately 90–91%, 72–82%, 54–68%, and 40–44%, respectively. The rates were similar to the training accuracy rates. Furthermore, the testing accuracy rate exceeded 90% only when the number of grades was 5. Therefore, the stipulated requirements could be met only when five grades were used; the z-score did not considerably influence the testing accuracy. Therefore, we conclude that the number of grades has a stronger effect on the accuracy of MLP model predictions than the z-score. In this study, the highest accuracy in predicting the width of the IC tray was achieved when the number of grades was 5; the average testing accuracy rate was more than 90%.

**Figure 8:**
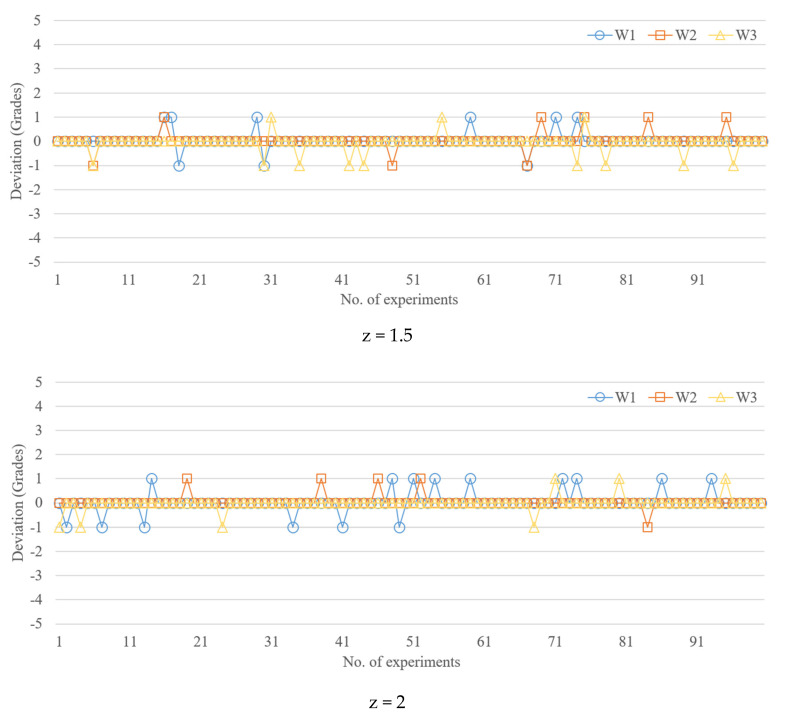

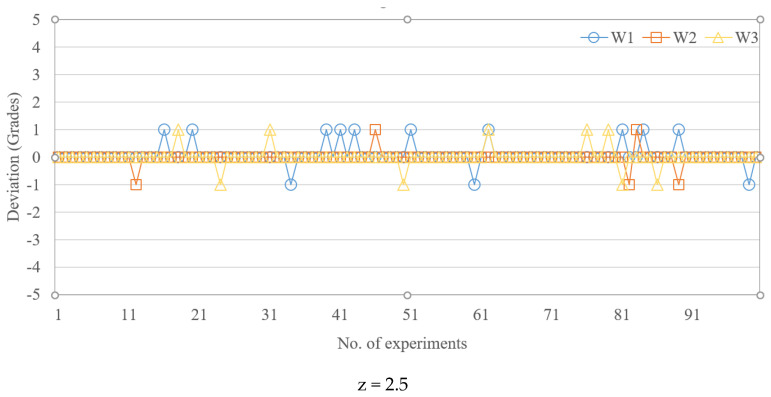
Comparison of actual and MLP-model-predicted grades (number of grades = 5).

Figure 8 shows width quality deviations observed for 100 test samples, each of which had the three widths; the number of grades was 5. At z-scores of 1.5, 2, and 2.5, the maximum deviation was 1 grade, which was approximately 35 µm. Therefore, the z-score did not have a clear influence on minimizing the prediction deviation. Table 8 shows that for 10 grades, when z-scores are 1.5, 2, and 2.5, the maximum deviations are 3, 1, and 4 grades, respectively. Specifically, a z-score of 2 was associated with the best performance in terms of prediction deviation (i.e., one grade, 17.7 µm). Similarly, for 20 grades, a z-score of 2 was associated with the best performance in terms of prediction deviation (i.e., four grades, 35.6 µm). For 50 grades, a z-score of 2 provided the best performance in terms of prediction deviation (i.e., 10 grades, 35 µm). In summary, the z-score (two, in this case) has a considerable impact on prediction accuracy.

### 4.2. Quality Assessment

**Figure 9:**
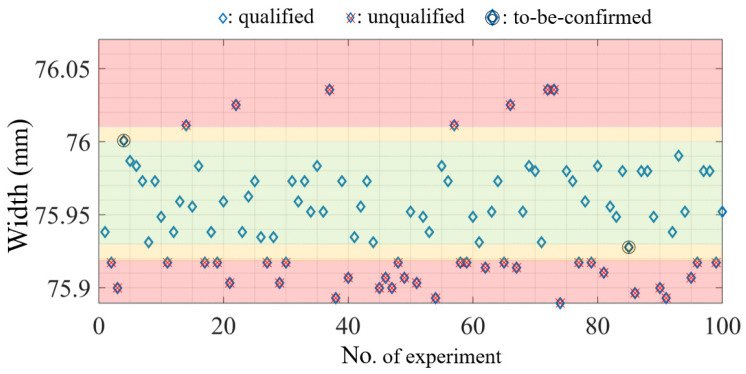
Areas of “qualified” (in **green**), “unqualified” (in **red**), and “to-be-confirmed” (in **yellow**) for W1.

Despite the high accuracy in quality prediction achieved using the proposed MLP model, 100% confidence cannot be guaranteed. Specifically, the quality of parts may not be judged correctly, resulting in manufacturing waste. Accordingly, we designed a narrow “to-be-confirmed” area, which is located between the “qualified” area and the “unqualified” area; we recommend predicting the quality of this area by using the MLP model in the future. Such a process would help prevent errors in judgment; however, such a process does not considerably reduce the burden placed on quality control operators. In this study, two “to-be-confirmed” areas were designed, each with a range of 0.01 mm. Consider, for example, W1 in Figure 9; when 50 grades and a z-score of 2 were considered, the specification limits for the “to-be-confirmed” areas were 76.00–76.01 and 75.92–75.93 mm. Overall, 98 of the 100 test datasets were successfully judged as “qualified” or “unqualified.” However, in the “to-be confirmed” area, two of the 100 test datasets required further confirmation regarding their quality levels. Specifically, the operator must confirm their quality. In industries, the quality of only a few datasets must be confirmed.

## 5. Conclusions

In this study, we developed an automatic and rapid quality inspection system based on a feedforward MLP ANN model. To test the model, we conducted experiments on the injection molding of an IC tray. Four highly related quality indices extracted from system and cavity pressure curves were used as input data, and the three geometric widths of the manufactured part were used as output data. To obtain a successful training rate in the MLP model, we filtered outliers in the pressure curve using the standard score (z-score). In addition, each actual width was converted into various numbers of grades. The representative dataset was generated through a two-factor full-factorial experiment, which provided sufficient information under actual injection molding conditions. The results are summarized as follows:

(1) The use of quality indices in MLP model training could reduce the size of data, thus improving the training time. *Ph_index_* and *PI_index_* were extracted from the system pressure curve, and *Pp_index_* and *Pr_index_* were extracted from the near-gate and far-gate cavity pressure curves, respectively. According to the PCC, these four indices were highly related to the quality of the part widths; therefore, they helped to reduce the number of nodes and layers in the hidden layers. In the case study for predicting the width quality of the IC tray, the MLP model contained an input layer of four nodes, two hidden layers (one with 100 neural nodes and the other with 75 neural nodes), and an output layer whose number of nodes depended on the number of grades under consideration. Approximately 400 datasets (1600 datapoints) were used for model training, which yielded accurate learning results rapidly (the training time and the testing time are less than 10 min and 0.003 s, respectively);
(2) Both the number of grades and the z-score had a considerable influence on the accuracy of quality prediction. In model training, the average prediction accuracy rates observed when 5, 10, 20, and 50 grades were used were approximately 92–94%, 75–83%, 58–71%, and 50–55%, respectively. Initially, the average training accuracy range for each z-score decreased as the number of grades increased. When the number of grades was low, the z-score did not have a considerable impact on training accuracy. However, because the use of more grades can provide more categories of part quality, we conclude that the z-score helped in improving the training accuracy.
(3) In the prediction of the widths of the IC tray, a z-score of 2 provided the best performance in terms of prediction deviation, that is, 1 grade (17.7 µm). For 20 grades, a z-score of 2 provided the best performance in terms of prediction deviation, that is, four grades (35.6 µm). For 50 grades, a z-score of 2 provided the best performance in terms of prediction deviation, that is, 10 grades (35 µm). In summary, the z-score (z = 2 in this case) had a considerable impact on prediction accuracy;
(4) This study also proposed the concepts of “qualified,” “unqualified,” and “to-be-confirmed” for evaluating part quality using the MLP model. The user-defined “to-be-confirmed” area was located between the “qualified” area and “unqualified” area; performing additional measurements to confirm the MLP model results is worthwhile. Therefore, the prediction of quality is more accurate when very few datasets (instead of all datasets) need to be verified. This method is suitable for use in industries.

## Figures and Tables

**Table 1:** Injection molding process parameters.

Item		Unit	Parameters
Melt temperature		°C	205
Mold temperature		°C	60
Backpressure		MPa	4.5
Clamping force		Tons	70
Decompression on stroke		mm	10
Holding speed limit		mm/s	80
V/P switchover position		mm	12.45
Cooling time		s	16
Holding pressure	1st stage	MPa	40, 50, 60, 70, 80, 90, 100
	2nd stage	MPa	5
	3rd stage	MPa	15
Holding time	1st stage	s	1
	2nd stage	s	4
	3rd stage	s	5
Injection speed		mm/s	40, 50, 60, 70, 80, 90, 100, 110, 120

**Table 2:** Pearson correlation coefficients (PCCs) between geometric widths and quality indices.

Quality Indices	Sensor Position	Symbols	Pearson’s Correlation Coefficient		
			W1	W2	W3
$Ph_{index}$	System pressure		0.96	0.96	0.97
$PI_{index}$	System pressure		0.79	0.81	0.78
$Pp_{index}$	Near the gate	SN1	0.95	0.96	0.96
$Pr_{index}$	Far from the gate	SN2	0.93	0.93	0.92

**Table 3:** Number of outliers versus z-score.

z Score	Number of Outliers		
	W1	W2	W3
2.5	0	0	0
2	10	15	11
1.5	52	50	52

**Table 4:** Bandwidth versus various numbers of grades and z-scores (unit: µm).

Grade Number	Standard Score, *z*		
	z = 1.5	z = 2	z = 2.5
5	34.8	35.4	35.4
10	17.4	17.7	17.7
20	8.7	8.9	8.9
50	3.5	3.5	3.5

**Table 5:** Hyperparameters of MLP model.

Item		Parameter
Software and version		Python 3.6.9
Loss function		Categorical Crossentropy
Optimizer		Stochastic Gradient Descent
Learning rate		0.49
Activation function		Sigmoid function, Softmax function
Metrics		Accuracy
Batch size		10
Epoch		10000
No. total dataset		352~404 (depends on the z score value)
No. testing dataset		100
No. neural node of:	Input layer	$Ph_{index}$, $PI_{index}$, $Pr_{index}$, $Pp_{index}$
	1st hidden layer	100
	2nd hidden layer	75
	Output layer	5, 10, 20, 50

**Table 6:** Average training accuracy versus z-score and number of grades.

Grade Number	Average Training Accuracy (%)		
	z = 1.5	z = 2	z = 2.5
5	94	93	92
10	75	83	80
20	58	71	66
50	55	50	53

**Table 7:** Average testing accuracy versus z-score and number of grades.

Grade Number	Average Training Accuracy (%)		
	z = 1.5	z = 2	z = 2.5
5	90	91	91
10	72	80	82
20	54	68	62
50	44	40	41

**Table 8:** Relation of average, standard and maximum deviation in three width, z-scores and grades.

Unit: Grade										
		Average			Standard Deviation			Maximum Deviation		
		W1	W2	W3	W1	W2	W3	W1	W2	W3
Grade 5	z = 1.5	0.09	0.08	0.12	0.29	0.27	0.33	1	1	1
	z = 2	0.15	0.05	0.07	0.36	0.22	0.26	1	1	1
	z = 2.5	0.13	0.05	0.07	0.34	0.22	0.29	1	1	1
Grade 10	z = 1.5	0.29	0.23	0.35	0.59	0.42	0.48	3	1	1
	z = 2	0.27	0.13	0.19	0.45	0.34	0.39	1	1	1
	z = 2.5	0.23	0.13	0.24	0.42	0.42	0.57	1	3	4
Grade 20	z = 1.5	0.88	0.56	0.6	1.16	0.83	0.74	5	4	2
	z = 2	0.68	0.32	0.32	0.89	0.58	0.6	4	2	2
	z = 2.5	0.9	0.47	0.35	1.06	0.85	0.66	5	4	3
Grade 50	z = 1.5	1.73	3.07	1.13	2.57	3.83	1.44	14	17	5
	z = 2	1.75	1.27	1.59	2.19	1.64	2	12	10	6
	z = 2.5	1.74	1.71	1.59	2.41	2.35	2.15	15	12	11

## Data Availability

Data can be provide on request.
